# Lived experiences of families of meningitis patients and survivors in the Upper West Region of Ghana

**DOI:** 10.1371/journal.pgph.0002894

**Published:** 2024-11-27

**Authors:** Damien Punguyire, Ambrose Naawa, Linus Baatiema, Simon Aabalekuu, Munawar Harun Koray, Avevor Patrick Mawupemor, Sally-Ann Ohene

**Affiliations:** 1 Ghana Health Service, Upper West Regional Health Directorate, Wa, Ghana; 2 Centre for Environment, Migration and International Relations, Simon Diedong Dombo University of Business and Integrated Development Studies, Wa, Ghana; 3 School of Public Health, Faculty of Public Health, University of Port Harcourt, Port-Harcourt, Nigeria; 4 Southern Medical University, Guangzhou, China; 5 World Health Organisation, Country Office, Accra, Ghana; PLOS: Public Library of Science, UNITED STATES OF AMERICA

## Abstract

Meningitis is a fatal condition and survivors often face long-term effects and often burdened their families. It is therefore important to understand how families cope with the aftermath of the infection. This study examined the experiences of families of meningitis patients and survivors in Ghana’s Upper West Region to support public health interventions. The researchers employed a Giorgi phenomenological qualitative method to conduct the study between March and April 2023. Data were collected from 40 facilities and four District Health Directorates. Sixteen participants, including survivors and parents, were selected from meningitis linelists. The study employed content analysis, with a coding system revealing five main themes. Inter-coder reliability was checked, and peer debriefing was used to ensure credibility. The study identified five main challenges: reduced productivity, inability to perform labor-intensive work, financial strain on households, psychological trauma, and the impact of social support. These experiences highlight the subjective nature of post-meningitis challenges and are consistent with existing literature. Meningitis survivors and their families face physical, emotional, and financial challenges, which can have long-term impacts. However, social support plays a crucial role in resilience and recovery. The study recommends that health institutions establish follow-up programs to monitor long-term effects on survivors.

## 1. Introduction

The meninges are the membranes that cover the spinal cord and the brain. Meningitis occurs when there is an infection of the meninges. It is a critical public health concern among health practitioners and affected communities [1]. Several pathogens cause the disease. The common ones are bacteria, fungi and viruses. Nonetheless, bacterial meningitis is the commonest with *Streptococcus pneumoniae*, *Neisseria meningitides* and *Haemophilus* influenza accounting for about 80% of all bacterial meningitis [2]. The annual estimate of bacterial meningitis globally is 1.2 million. Case-fatality rates and incidences differ by age categories of exposed or susceptible populations, causative pathogens, regions and countries [3]. Though meningitis affects persons of all ages, younger age groups are more at risk [3].

The transmission of the causative pathogen is from one individual to another via throat secretions or respiratory droplets from carriers [1]. The incubation period is 4 days but can take between 2 and 10 days [4]. Meningitis may occur within a wide spectrum of contexts ranging from small clusters, sporadic cases, to global epidemics with associated cyclical variations. In the absence of treatment, case fatality rate can rise to 70% with 10%-20% of survivors living with permanent sequelae such as mental retardation, hearing loss, and non-functional limbs [1, 2]. Beyond these, studies have also reported undesirable psychological stresses meningitis survivors go through. Psychological concerns such as depression, anxiety, post-traumatic stress disorder and stigma among others are some of the commonly reported consequences that affect the quality of life of survivors [5, 6].

Meningitis is common in sub-Saharan Africa, particularly within the meningitis belt, which spans from Senegal in West Africa to Ethiopia in East Africa [1]. It is worth noting that Ghana lies within the Africa meningitis belt, the region that accounts for the highest meningitis burden globally [7]. Routine immunization programs and ensuring high immunization coverage are important steps to overcome the resurgence of meningitis epidemic [1]. WHO and the global community have instituted interventions to halt meningitis. For instance, the WHO in collaboration with several partners developed the global roadmap “Defeating Meningitis by 2030” [1]. The roadmap outlines the global vision “towards a world free of meningitis” irrespective of the cause.

In spite of the global commitment in the fight against meningitis, it still ravages countries within the meningitis belt including Ghana. More than 400 meningitis deaths and 3,000 meningitis cases were recorded in Ghana between 2010 and 2015 [8]. Within Ghana, the Upper West region is one of the five regions that lie within the meningitis belt [9]. A review of meningitis cases in the region between 2009 and 2013 revealed 980 (52%) cases were females. Case fatality rate was 12.2% with 14 deaths per 100,000 population [8].

Despite the national and global interest in combating meningitis, there is the absence of knowledge of the lived experiences of families of meningitis patients and survivors in the Upper West Region to guide interventions. A review of the literature on the lived experiences of meningitis patients in other locations showed that there are some psychological and economic adjustments in the lives of meningitis survivors and their families [10]. Family support during the sickness and recovery phases of meningitis patient is very crucial. Feeding, adherence to medication schedule, patient hygiene, infection prevention and control among others are some of the major areas of support from family members to victims. Family members also provide financial support in buying medications and offsetting other costs [11, 12].

Considering the possible consequences of meningitis on victims and their families, an understanding of their lived experiences may be useful in directing tailored public health interventions to reduce the intensity of the impact on the lives of families of victims and survivors. Consequently, the study investigated the lived experiences of families of meningitis patients (either dead or alive) and survivors of the disease in the Upper West Region, to inform appropriate public health interventions.

## 2. Methods

### 2.1 Study design

The study utilized Giorgi’s phenomenological study design to comprehensively investigate and evaluate the lived experiences of both parents and survivors affected by meningitis in the Upper West Region. Following a qualitative approach, themes and sub-themes were derived from the data collected through interviews. The adoption of Giorgi’s phenomenological design was deliberate, as it facilitated a deep exploration of the perspectives of both survivors and their families, allowing for the development of meaningful themes grounded in the participants’ experiences. It is important to note that the study’s sole objective was to document the lived experiences of meningitis survivors and the perspectives of their families with the aim of providing a rich and nuanced understanding of their encounter with the disease.

### 2.2 Study setting

The study took place in four districts of the Upper West Region of Ghana. The area lies directly on the African meningitis belt. The study took place between March and April 2023 covering 40 health facilities and 4 District Health Directorates. The Health Directorates are Jirapa, Lawra, Nandom and Nadowli Kaleo. The four districts were the target of the study because of their consistent recording of high meningitis cases over the years.

### 2.3 Study participants

By purposive sampling method, survivors of meningitis and caregivers of victims—whether dead or alive—in the four sampled districts noted as the hub of meningitis in the region participated in the study. The inclusion criteria included; 1) should be a survivor of meningitis and resides within the area chosen for the study 2) should be a parent/guardian or a caregiver or relative who supported the survivor or deceased during the affected moments.

The study team obtained the line list of meningitis cases from the districts with the assistance of District Disease Control Officers, Community Health Officers and Community Health Volunteers. With the aid of the line list data, the team identified the cases one by one for interviews. The team interacted with eight survivors and their families, interviewing 16 participants in all. The research officers did not encounter any instances where a family or survivor declined the interview. All targeted participants willingly consented to be interview to the interview.

### 2.4 Data collection instrument

The data collection instrument employed for this study consisted of face-to-face semi-structured in-depth interview conducted separately for survivor and their familiars at times and locations convenient for them. To develop the semi-structured interview guide (S1 Text), the team undertook a rigorous process that involved a review of existing literature and consultation with experts. This approach allowed us to create a comprehensive guide that encompassed key questions and prompts aimed at eliciting detailed information of survivors and families’ experiences during and after the sickness. The semi-structured interview guide, tailored for both survivors and family members, included a set of predetermined questions and prompts. These guided the conversation, ensuring coverage of essential aspects, while also allowing for flexibility and spontaneity in exploring participants’ unique perspectives. Additionally, follow-up probes and prompts were incorporated to delve deeper into participants’ experiences, opinions, and viewpoints. Regarding the validity of the instruments, expert judgment was sought through literature reviews and consultation with professionals experienced in the field of meningitis and qualitative research. This process aimed to establish content validity by ensuring that the interview guide effectively captured the relevant aspects of survivors and families’ experiences. The experts provided critical insights and recommendations, contributing to the refinement of the interview guide to enhance its appropriateness and relevance. As for the analysis of validity, we focused on content validity, emphasizing the relevance, representativeness, and clarity of the interview guide in capturing the lived experiences of survivors and their families. The instruments were self-developed through a meticulous process involving both literature review and expert input, ensuring a robust foundation for data collection. To maintain the rigor of the study, all interview sessions, lasting between 30 to 45 minutes for both survivors and family members, were audio-recorded with participants’ permission. The recordings were transcribed verbatim in the participants’ language before the next interview session, ensuring accurate representation of the participants’ narratives in subsequent analyses.

### 2.5 Data analysis and management

The team used Giorgi’s phenomenological analysis to understand the experiences of family members and survivors of meningitis in the Upper West Region of Ghana.

The Giorgi’s phenomenological study design was useful in assisting the team to comprehensively investigate and evaluate the lived experiences of both parents and survivors affected by meningitis in the Upper West Region. Following a qualitative approach, themes and sub-themes were derived from the data collected through interviews. The adoption of Giorgi’s phenomenological design was deliberate, as it facilitated a deep exploration of the perspectives of both survivors and their families, allowing for the development of meaningful themes grounded in the participants’ experiences.

Giorgi’s qualitative phenomenological study is conducted in multiple steps. The first step involves qualitative data collection, typically through in-depth interviews. Participants provide detailed description of their experiences in relation to the research topic. The next step involves the researcher setting aside their biases to engage with the data more objectively and extracting invariant themes across the data. This is followed by a thematic analysis and finally synthesizing the themes into coherent a description that captures the essence of the phenomenon as experienced by the study participant.

The approach underscores the significance of setting aside preconceived notions and prioritizing participants’ own words. Researchers using Giorgi’s approach engage a process of coding, categorizing, and analyzing data to reveal the underlying meanings and structures that constitute participants’ experiences [13].

Data collected from the field was transcribed in English for the organization of study data and codes. Data were initially analyzed and independently coded by two authors, LB and AN, to identify and agree on emerging themes. The team compared codes for refinement and clarification. Observed differences were resolved through group discussions. Through repeated examination, an iterative review of the themes, and grouping of codes, the team identified prevailing themes that represented experiences of family members and survivors of meningitis. The team discussed and selected quotations that illustrated the themes and continually monitored for data saturation. The analyses indicated the achievement of data saturation.

Trustworthiness of the data followed strategies proposed by Lincoln and Guba in [14] as cited in Polite & Beck, [15]. All interviews were carried out by experienced research assistants who are experts in conducting qualitative research and interviewing techniques. Nonetheless, they were still trained in data collection. Finally, sufficient numbers of interviews were performed to ensure the saturation of concepts. At the end of the data collection, the transcripts were sent for peer checking by four experienced qualitative researchers in social sciences and field epidemiology as well as disease control officers to review the credibility of the extracted themes and sub-themes. Finally, in addition to audio-records and transcripts, multiple data sources, including field notes and observation were used.

### 2.6 Ethical considerations

The Ghana Health Service Ethics Committee reviewed and gave approval for this study (GHS-ERC:011/10/22). Prior to each interview, all participants provided written informed consent. Participants were not compensated or given motivating materials in exchange for their participation. All interviews were audio-recorded with the consent of all participants. Those without writing abilities thumb printed. Participants under the age of 18 had to ask their parents, or other guardians for permission.

## 3. Results

### 3.1 Demographic characteristics of participants

Respondents were asked about their socio-demographic characteristics. The ages of the respondents range from 21 to 60 years. The majority (N = 8, 50%) of the respondents were in their 40’s to 50’s with few (N = 6, 31.25%) of them found within the 30’s and 20’s. More than half (N = 9, 56.25%) of them were married and staying with their partners. Of the 16 people who were interviewed, 7(43.75%) of them were unemployed. As seen in table S1 Table.

### 3.2 Parents and survivors experience of meningitis

This section presents the major experiences shared by survivors of meningitis and their caregivers following their recovery. They revealed varied experiences and this reinforces the subjective nature of the feeling and experiences noted in the literature. Five main unique but interrelated dimensions emerged from the interviews. These are, reduced productivity, inability to do any hard work, financial pressure on household following the incident, psychological impact and family support.

### 3.3 Reduced productivity

Increased productivity is one of the key pillars for boosting household income when it comes to footing bills, expanding business and supporting the socio-economic development of families as was shared by survivors of meningitis. Survivors recounted loss of productivity after contracting meningitis and some could not catch up with their colleagues in the market. Some indicated they had lost their stable jobs. The parent of one survivor had this to say:

*Our daughter’s work output has drastically reduced*. *Since she recovered*, *she no longer goes to farm like she used to do*, *she can no longer under-take house chores like she used to do among other life duties*. *Any time she attempts to do any of these things*, *we end up in the hospital and so we decided that she stays off house chores and farm activities*. *This new development puts a lot of pressure on the mother and I.*

*(* ***Father of a survivor*, *aged 58)*.**

The uncle of a survivor added:

*The sickness didn’t really happen at a good time*. *He was admitted in the farming season for two weeks*. *We had to find time to visit him and provide his needs*. *It really affected our farming activities*. *I farmed small but he who is the strong man couldn’t farm at all on his own*. *Even funerals within the period*, *we couldn’t attend*

***(Uncle of a survivor*, *aged 43)*.**

### 3.4 Psychological impact

The field interviews revealed that the psychological impact of meningitis was a major problem to most survivors and their families. Overall, the psychological fragility after experiencing meningitis has serious consequences on the individual and family in their social relations. Phobia, insecurity to one self, social isolation, feeling of difference compared with others and feeling of being misunderstood were some of the psychological concerns raised by both survivors and family members. A survivor, while in uncontrollable tears had this to say:

*“Okay*, *I’m not sure I’m comfortable with the question you are asking*. *Life has indeed been terrible following my discharge*. *Before I was infected with the condition*, *I was pregnant and had to be detained in the hospital for almost two months of hard stress and intensity*. *I was finally discharged*, *came home*, *and always felt weak and dizzy*. *I eventually went into labor*, *suffered in there*, *only for my husband to come in and the doctor telling me that the baby wasn’t staying and they needed to save me and the baby*. *So*, *the doctor said he wanted to run some procedures*. *This wasn’t well communicated to me*, *and I consented only when I recovered*, *and they were telling me my womb was turned so that I cannot give birth*. *This made me feel bad till date*. *How can a woman’s womb be blocked? Was it because of the sickness that made them block my womb? What happens if my husband should divorce me? Where do I have to start from? In fact*, *this cerebrospinal meningitis has really caused me a lot of pain*, *and I can never forget that till I die”*

*(****Survivor*, *aged 35****)*.

A housewife who survived also narrated as follows:

*“n bie (meaning my son) am a new person following my survival from this sickness*. *I now can’t socialize like I used to do*, *I sit as if I have no body in my life*. *I have five children all of them too are in the south*. *It has come to be like*, *if not my women group members who sometimes come to visit*, *my only interaction rests on my husband who is equally not the talking type*. *In fact*, *this sickness reminds me of a proverb by my grandfather which says that “it is only when you are confronted with a problem that you know your loved ones” but this sickness has really brought me to the reality of the proverb”*

***(Survivor*, *aged 58)*.**

A caregiver had this to add:

*“I was scared when they were taking her sample*. *I was asked to go and buy some medication for her and when I returned*, *they had used something (screen) to cover her and I thought she had died*. *As such*, *I screamed and they stopped me and told me she was not dead*. *They were taking her sample to do lab test”*. *I have since not been comfortable particularly her frequent complains anytime she engages in any stressful work.”*

***(Caregiver of a Survivor*, *aged 44)*.**

### 3.5 Financial pressure on households

The study revealed that affordability of hospital bills and the cost of feeding victims are critical issues as far as care for the survivors is concerned. Participants indicated that the care they received from the care healthcare providers was very expensive. This was what one of the participants had to say:

*“the incident happened at a time we didn’t have money at home*. *I was in the farm when I was called that our daughter fell and could not walk*. *I wasn’t having motorbike*. *I bought fuel into someone’s motor and we rushed her to Jirapa hospital where she was detained for three months*. *During her admission*, *lab tests were run and all this was costly*, *it wasn’t easy*. *We could buy medicine for two–three times before the day will end*. *Hmmmmmm*, *we were actually not laughing there*. *We were financially down but we have no choice because our daughter’s life was at stake”*

***(Stepmother of a survivor*, *aged 37)*.**

Another survivor’s relative said:

*“Following our brother’s recovery*, *we were happy*. *However*, *we now struggle to put food on the table because we have almost depleted the little*, *we were depending on in order to cover medical bills and daily transportation of food to the hospital*. *The situation has led to significant indebtedness in our household*, *to the point that we are not trusted within the community*. *We had to resort to borrowing to fulfill our financial obligations and meet other needs.”*

***(Brother of a Survivor*, *aged 29)*.**

One of the survivors’ mothers had this to add:

*“it’s not everything we can say*. *Paying for hospital bills when my daughter was hospitalized was difficult and even some of the prescribed medicines*. *We battled ourselves left right till we had all those things cleared*. *Even when they discharged her and we couldn’t clear all the bills*, *the nurses didn’t allow us to go until the bills were fully paid”*

***(Mother of a Survivor*, *Aged 50)*.**

A survivor revealed the following:

*“We really went through a lot when I was admitted*. *The very one that got me almost crying was the difficulty my people were going through to transport food to the hospital on a daily basis because we couldn’t afford the cost of fuel for daily trips*. *It was a distressing time for us as we contemplated life and death”*

***(Survivor*, *Aged 38)*.**

### 3.6 Limited ability to perform harder tasks

The study showed that Survivors of meningitis in the Upper West Region grapple with challenges that limit their ability to perform hard tasks. These challenges include physical and cognitive disabilities and emotional distress. Some of the opinions gathered through the field interviews are as follows:

*“hmmmm it has really not been easy*. *I was actually processing shea butter to sell for a living as well as selling “Koosee” and assisting my husband to farm but now the strength is not there*. *I am no longer able to bend down for long because of waist pain*. *So*, *the quantity of work I used to do has drastically reduced hence affecting my productivity and source of livelihood”*

***(Survivor*, *aged 48)*.**

A farmer added:

*“The impact of meningitis weighed heavily on the patient’s ability to perform major tasks*. *The condition represented a significant barrier in his daily life*, *affecting his productivity*, *independence*, *and overall sense of well-being*. *He was virtually farming to feed us*, *unfortunately*, *he went down completely and could not support us in farming again*, *even though he recovered*. *His work output has drastically changed*. *Someone who used to farm and would harvest close to 50 bags of maize and around 10 bags of groundnut*, *is now struggling to produce even a single bag of maize or groundnut*. *In fact*, *we have suffered significant losses.”*

*(****Uncle of a survivor*, *aged 52****)*.

An aunty also shared that:

*“My son’s happiness has disappeared since he was deeply affected by meningitis*. *Now*, *when two or more people are sitting together*, *he prefers to isolate himself and just watch*. *This alone has put the family in a disorganized state*. *Currently*, *we simply don’t know what pleases him*. *Hmmmmm*. *That is Life!”*

*(* ***Uncle of a survivor*, *aged 48)*.**

A survivor added:

*“It’s been a journey*, *you know? Dealing with cognitive hiccups feels like my own mind playing tricks*. *The emotional toll? Heavy–frustration*, *self-doubt*, *it’s all there*. *Now*, *the harder tasks aren’t just tasks; they’re massive obstacles*. *Not that I don’t want to tackle them*, *but cognitive glitches team up with emotional baggage*, *making even simple things feel like climbing a mountain*. *So*, *yeah*, *grappling with this cognitive-emotional task constraint.”*

***(Survivor*, *Aged 38)*.**

### 3.7 Family support

Beyond the financial support family members had to provide when their relatives were down with meningitis, a number of survivors were also excited about the social and moral support they got from family members during those trying times. A survivor said:

*Family support was massive*. *If not for my family*, *I would have died*. *I don’t really have friends like that at home because am not based here*. *If it was Afram Plains*, *I could tell their level of support*

***(Survivor*, *Aged 59 year)*.**

An educated caregiver had this to share:

*“It is important to recognize that family support is not limited to the immediate aftermath of the illness but extends throughout the entire recovery process*. *Meningitis comes along with long-term effects on the patient’s physical and cognitive abilities*, *and ongoing family support helps the individual navigate these challenges and adapt to any changes in their daily lives*. *If not for our support*, *my brother would have passed on by now*. *We had to rush him to the hospital and we have since been around encouraging him morally and physically supporting him”*

*(****Caregiver of a Survivor*, *aged 41****)*.

## 4. Discussion

The aim of the study was to investigate the lived experiences of survivors and caregivers of meningitis victims in the Upper West Region to inform appropriate public health interventions. The findings revealed varied experiences categorized into five main unique but interrelated dimensions: reduced productivity, financial pressure, family support, inability to do any hard work and lastly psychological impact.

One of the most commonly reported challenges among survivors of meningitis is reduced productivity. Survivors disclosed that the sickness took so much of their time and that of family members when they were on admission in hospital. Given that the main occupation of the family members of most survivors is farming, they lost many productive hours during the hospitalization phase of the disease. Many of them also noted that, even after discharge from hospital, they were not able to perform tasks they could previous do with ease. Even more, in view of the fact that most survivors were the breadwinners of their families, their impaired ability to work results to decreased productivity and this has far-reaching consequences for the well-being of their families. This finding is consistent with previous research conducted in Denmark, United Kingdom and a systematic review, which has shown that survivors of meningitis often experience long-term disability, reduced quality of life and productivity [16–18]. It is worth noting that the experience of meningitis survivors can vary widely depending on factors such as the severity of their illness, age, pre-existing health conditions, and access to medical care and social support. Therefore, while reduced productivity may be a commonly reported challenge, it is possible that some survivors may not experience this particular difficulty or may prioritize other challenges as more significant. Early reporting is therefore a key measure to avoiding death, minimizing the effect of meningitis including permanent retardation. Early diagnosis will lead to early and appropriate treatment.

The study also showed that meningitis victims usually suffer physical challenges, including fatigue and general body weakness. These effects can limit their ability to perform physical activities that are a bit rigorous and even if they previously performed them routinely. All the survivors interviewed complained of sharp pains in their spine where the sample was taken. Some indicated their inability to sit or bend for long, among others. As such, survivors now struggle to do manual tasks such as shea butter processing and farming which they used to do with ease. The findings are consistent with the arguments of Emery et al. [19] who highlighted the management of common clinical problems experienced by survivors of cancer and provided insights into the challenges faced by individuals who have undergone cancer treatment and the potential long-term physical and cognitive effects. The authors noted that cancer survivors often experience a range of physical and psychological issues, including fatigue, pain, anxiety, and depression. These challenges can significantly impact a survivor’s quality of life and may also limit their ability to perform daily activities and work. As part of the management of such cases, clinical psychologist should be assigned to them to help counsel and integrate them back into society.

Financial pressure on households following the incident emerged as one of the key findings from the study. Families recounted the financial pressures they had to go through due to the high cost of medical care. Even worse, most victims did not have an active health insurance subscription to guarantee them access to care without directly paying for the services. This is consistent with prior scholarly work on the subject especially the findings of Schiess, Groce, and Dua [20], that noted meningitis could have a significant economic impact on individuals and society as a whole. Specifically, their cost of treatment, lost productivity, and long-term disability can impose a heavy financial strain on affected families and communities. The situation is particularly dire as stated under reduced productivity that the economically viable individuals are the people often affected by meningitis [21]. It limits their ability to mobilize financial resources to support hospital bills. The social support system should have presented an opportunity to support families financially during difficult times however, as indicated by one of the family members of a survivor, the only support they got from other family members was in kind. In fact, many of these families were somewhat equally battling with different degrees of financial burdens. Following these useful lessons, we use the opportunity to recommend that everyone should endeavor to have an active NHIS card and observe the meningitis prevention protocols to avoid infection.

The study found that family support was a crucial dimension. Parents of meningitis patients and survivors who participated in the interviews reported giving or receiving some support. Areas of support included feeding, bathing, running daily errands, reassurance of quick recovery, counselling, and monetary support. This support was critical in helping survivors cope with the physical, emotional, and financial challenges of their illness. Zhang et al. [22] highlighted the importance of social support in promoting resilience and recovery among survivors of meningitis. According to Olbrich, et al. [23] and Bhadhuri, et al, [24] social support, particularly from family members, is essential in promoting health and well-being in individuals facing health challenges. It is therefore important to conduct massive health education on meningitis especially during the meningitis season and in endemic areas to educate people on how to prevent, manage and the needed family support when one is affected. The role of family support is so principal and needs to be enhanced. Leveraging on community traditional structures presents an opportunity for awareness creation and adherence to meningitis safety protocols.

Finally, the study revealed the undesirable psychological impacts meningitis survivors go through Some of the commonly reported psychological concerns included depression, anxiety, post-traumatic stress disorder, and other mental health conditions. A family member of a survivor indicated how her daughter struggles with isolation and social withdrawal, as she feels stigmatized or misunderstood by her colleagues in school. Survivors equally recounted how they lost some of their close associates during and after the sickness. They felt at risk of contracting the disease if they visited them. This is in unison with [10, 25]. They reported in their study that meningitis could have significant psychological consequences for survivors and their families. As part of the management of such cases, clinical psychologist should be assigned to them to help counsel and integrate them back into society.

### 4.1 Strengths and weaknesses of the study

This research provided insights into the personal experience of meningitis survivors as well as their families. It delved into their work and other activities beyond the sickness. It further explored the critical role of relatives in restoring their quality of life. Our study, to the best of our knowledge, is the first to use a validated methodology to explore the experience of acute bacterial meningitis survivors in the Upper West Region of Ghana.

The study reported the subjective responses of survivors of meningitis as well as their families. Further research could be conducted to verify the cause of the pains in their spines and cause of the stigma and social isolation.

## 5. Conclusion

The findings point to the fact that survivors of meningitis face a range of physical, emotional, and financial challenges during the illness and post-recovery. These challenges are often interrelated and can have long-term impacts on the survivor and their family members. However, social support and warm reception can also play a crucial role in promoting resilience and recovery. These findings highlight the need for support for individuals affected by meningitis. The study further underscores the indispensable role of family support to victims and recommends the need for further research and tailored interventions to reduce meningitis-related stigma. Further to that, it highlights the gaps in meningitis sequalae management and the need to institute programmes that ensure consistent follow-up that guarantees the restoration of the dignity and quality of life of survivors.

## Supporting information

S1 Text IDI guide. (DOCX)

S1 Table Demographic characteristics of participants. (DOCX)

S1 Data Study data-transcript. (DOCX)
