# Supplementary material for: Lived experiences of families of meningitis patients and survivors in the Upper West Region of Ghana
Source: PLOS Glob Public Health. 2024 Nov 27;4(11):e0002894. doi: 10.1371/journal.pgph.0002894 (PMC11602017; doi:10.1371/journal.pgph.0002894)
Supplement: S1 Text — (DOCX) [file pgph.0002894.s001.docx]

**Appendix 1**

**Lived Experiences of Families and Survivors of Meningitis in the Upper West Region, Ghana.**

Dear Participants,

Thank you for participating in this important qualitative study examining the Lived Experiences of Families and Survivors of Meningitis in the Upper West Region, Ghana. Your insights are invaluable in shedding light on the intricacies of this critical aspect of healthcare delivery. In this guide, we aim to capture the personal stories, challenges, and triumphs of individuals directly affected by meningitis in this specific region.

Meningitis, a potentially life-altering infection, not only impacts the diagnosed individuals but also leaves a lasting imprint on their families and communities. This IDI guide seeks to unravel the various dimensions of these experiences, delving into the emotional, social, economic, and psychological aspects faced by families and survivors. By engaging in conversations about day-to-day realities, coping mechanisms, and the socio-economic impacts of managing meningitis, we hope to gain a comprehensive understanding of the challenges faced by families and survivors. Additionally, we aim to uncover insights into the prevention and management of meningitis in community settings. Your participation in these interviews is crucial for amplifying the voices and perspectives of those directly affected by meningitis. By sharing lived experiences, we aspire to contribute to a deeper understanding of the human impact of meningitis in the Upper West Region and beyond.Thank you for your willingness to share your stories, helping us build a collective knowledge that can potentially improve the lives of individuals and families affected by meningitis.

Sincerely,

**Signed**

**Dr. Damien Punguyire (PI)
Regional Director of Health Services**

**Upper West Regional Health Directorate**

**Survivors of** Meningitis **Tool:**

1. Tell me about your experience when you were predisposed to Meningitis. Probe for:
   1. Please share your everyday experiences with meningitis when you were affected.
   2. Has the condition changed your daily life in any way?
   3. How has your life been affected?
   4. Describe the family roles you used to play then and now.
   5. Explain how the condition affected your ability to work.
   6. How have friends' and family members' attitudes toward you changed?
   7. Can you elaborate on how the condition affected your self-esteem?
2. Can you share with us some coping strategies you employed when you were infected with the condition?

**Family or Relative Tool:**

1. When your family member, friend, or neighbor was infected with meningitis, what were some of the challenges you faced in managing him/her daily? (Probe: Was it financially expensive? Did it consume all your time and energy, etc.)
2. What were some of the socio-economic implications associated with managing or assisting the meningitis patients?
3. What difficulties were encountered during treatment?
4. How do you manage meningitis in this community?
5. In your view, how can meningitis be effectively prevented in the community?
6. Is there anything else you would like to share about your experiences in assisting the victim?

Thank you.
