# Supplementary material for: Lived experiences of families of meningitis patients and survivors in the Upper West Region of Ghana
Source: PLOS Glob Public Health. 2024 Nov 27;4(11):e0002894. doi: 10.1371/journal.pgph.0002894 (PMC11602017; doi:10.1371/journal.pgph.0002894)
Supplement: S1 Table — (DOCX) [file pgph.0002894.s002.docx]

**Table 1: Demographic Characteristics of Participants**

| **No.** | **Participant (**pseudonym**)** | **Who participant lives with** | **Age** | **Occupation** | **Marital status** |
| --- | --- | --- | --- | --- | --- |
| 1 | P1 | Survivor | 40yrs | Unemployed | Divorced |
| 2 | P2 | Father of Survivor | 42yrs | Farmer | Widow |
| 3 | P3 | Survivor | 38yrs | Farmer | Married |
| 4 | P4 | Husband | 46yrs | Farmer | Married |
| 5 | P5 | Survivor caretaker | 56yrs | Unemployed | Married |
| 6 | P6 | Survivor Stepmother | 60yrs | Unemployed | Divorce |
| 7 | P7 | Husband | 35yrs | Farmer | Married |
| 8 | P8 | Survivor | 38yrs | Unemployed | Divorce |
| 9 | P9 | Survivor | 21yrs | Unemployed | Single |
| 10 | P10 | Survivor mother | 50yrs | Unemployed | Married |
| 11 | P11 | Survivor mother | 49yrs | Unemployed | Married |
| 12 | P12 | **Survivor** | 21yrs | Farmer | Single |
| 13 | P13 | **Survivor** | 19yrs | Student | Single |
| 14 | P14 | Parent | 45 | Farmer | Married |
| 15 | P15 | **Survivor** | 37 | Farmer | Married |
| 16 | P16 | Parent | 44 | Housewife | Married |

Source: Fieldwork, 2023
