# Supplementary material for: Lived experiences of families of meningitis patients and survivors in the Upper West Region of Ghana
Source: PLOS Glob Public Health. 2024 Nov 27;4(11):e0002894. doi: 10.1371/journal.pgph.0002894 (PMC11602017; doi:10.1371/journal.pgph.0002894)
Supplement: S1 Data — (DOCX) [file pgph.0002894.s003.docx]

Participants 1

**Interviewer:** **Thank you for participating in this interview. To begin, could you please tell me about your experience when you were predisposed to Meningitis?**

**Responses:**  don’t even know whwre to start. Meningitis was a profound experience for me, one that I wouldn't wish upon anyone. It began with feeling extremely weak and dizzy, which eventually led to multiple hospital visits. Every day felt like a struggle, and even the simplest tasks, like getting out of bed or preparing a meal, became monumental challenges. The uncertainty and fear surrounding my health added to the overwhelming nature of the situation.

**Interviewer:** **How did the condition change your daily life?**

**Responses:**  The impact of Meningitis on my daily life was significant and far-reaching. Before falling ill, I prided myself on being active and independent, managing both work and household responsibilities with ease. However, Meningitis stripped away that independence, leaving me reliant on others for even the most basic tasks. It was a humbling experience, but also one filled with frustration and a sense of loss.

**Interviewer:** **Can you describe how your life has been affected overall?**

**Responses:** Meningitis had a profound effect on every aspect of my life. Physically, I struggled to regain my strength and energy, often feeling fatigued and unable to engage in activities I once enjoyed. Emotionally, I experienced a rollercoaster of emotions, from fear and anxiety about my health to sadness and grief over the life I had lost. My relationships with family and friends also shifted, as they tried to navigate how best to support me during this challenging time.

**Interviewer:** **What were the family roles you used to play then and now?**

**Responses:**  Before Meningitis, I played a significant role in my family dynamic, often taking on the responsibilities of a caregiver and provider. However, after falling ill, those roles reversed, and I found myself in need of support and assistance from my loved ones. It was a difficult transition, both for me and for them, as we adjusted to this new reality.

**Interviewer:** **How did the condition affect your ability to work?**

**Responses:**  Meningitis completely derailed my ability to work. I was forced to take a leave of absence from my job, and even when I attempted to return, I quickly realized that I was not physically or mentally capable of performing at the level I once had. It was a devastating blow to my self-esteem and identity, as my career had always been a source of pride and fulfillment for me.

**Interviewer: Have you noticed any changes in the attitudes of friends and family members toward you?**

**Responses:** Yes, definitely. While some friends and family members were incredibly supportive and understanding, others seemed to struggle with how to interact with me post-illness. There were instances of pity or discomfort, which made me feel alienated and misunderstood. It was a difficult adjustment for everyone involved, and it strained some of my relationships.

**Interviewer: Can you elaborate on how the condition affected your self-esteem?**

**Responses:**  Meningitis dealt a significant blow to my self-esteem. Suddenly finding myself dependent on others for basic tasks and unable to fulfill the roles and responsibilities I once had left me feeling inadequate and worthless at times. It was a constant battle to remind myself of my inherent value and worth, despite the limitations imposed by my illness.

**Interviewer: Moving on, can you share some coping strategies you employed when you were infected with the condition?**

**Responses:** Absolutely. Coping with Meningitis was a journey in itself, and I relied on a variety of strategies to navigate the challenges. Seeking support from loved ones and healthcare professionals was crucial, as was finding outlets for self-expression and reflection, such as journaling or therapy. I also leaned heavily on my faith and spirituality, finding solace and strength in my beliefs. Connecting with other survivors through support groups or online communities provided a sense of camaraderie and understanding that was invaluable during this difficult time.

**Interviewer: Thank you for sharing your experiences and coping strategies with us. Your insights will be invaluable in understanding the impact of Meningitis on individuals' lives.**

Participants 2

**Interviewer:** Thank you for participating in this interview. Can you share your experience when you were affected by Meningitis?

**Responses:**Yes. Meningitis was a harrowing experience for me. I had an unusual feeling of fatigue and intense headache. This escalated to more severe symptoms like fever and confusion. I was admitted to the hospital and spent several weeks undergoing treatment and rehabilitation. The whole ordeal was frightening and overwhelming.

**Interviewer:** How did Meningitis change your daily life?

**Responses:** Meningitis turned my world upside down. I was a busy and active individual, juggling work, family, and social commitments with ease before the sickness. You won’t belief that I struggle to perform the simplest routine tasks I use to do. Even getting out of bed or preparing a meal became a challenge, and I found myself relying heavily on family members for support.

**Interviewer: Can you describe how your life has been affected overall?**

**Responses:** Meningitis impacted heavily on every aspect of my life. Like I mentioned, I struggle to perform basic and routine activities I use to do, often experiencing pain and fatigue. Again I battled with fear, anxiety, and depression as I grappled with the uncertainty of my health and the challenges of recovery.

**Interviewer: What were the family roles you used to play then and now?**

**Responses:** Before the sickness, I played an active role in my family upkeep. Now, most of those roles are performed by other family members. It was a humbling experience, but also one that brought us closer together as we navigated the challenges of my recovery.

**Interviewer: How did the condition affect your ability to work?**

**Responses:** I am a teacher, but following the sickness, I couldn’t go to school. Even though I was discharged from the hospital, I couldn’t go to school. I had challenges with my head and circuit supervisor, thinking I didn’t want to come to school and teach. Even when I attempted to return to work, I struggled to keep up with the demands of my job and ultimately had to make significant adjustments to accommodate my new limitations. It was a major headache explaining to my supervisors and I had to start work when I was not completely ok. I don’t pray to be down with this kind of sickness again.

**Interviewer: Have you noticed any changes in the attitudes of friends and family members toward you?**

**Responses:**Yes some changes have been observed. People are asking what kind of sickness it was considering the extent of my illness. Some seemed to expect me to bounce back to my old self overnight, while others were hesitant to acknowledge the severity of my condition. It was a challenging time, and I had to reach out to some of my friends to allay their fears. I must acknowledge that my family was very supportive, especially my immediate family.

**Interviewer: Can you elaborate on how the condition affected your self-esteem?**

**Responses:** Meningitis took a toll on my self-esteem in ways I never anticipated. You can’t imagine how, I had to depend on others for very basic tasks I use to perform. I struggled with feelings of guilt and shame for not being able to "bounce back" as quickly as I had hoped. It took time and a lot of self-reflection to come to terms with my new reality and learn to value myself.

**Interviewer: Moving on, can you share some coping strategies you employed when you were infected with the condition?**

**Responses:**Yes, like I indicated, I had to motivate myself couple with the family support and reassurance. I had to reach out to some of my friends, not all were positive but I found some support from most of them. And perhaps most importantly, I learned to be patient and compassionate with myself as I worked toward my recovery.

**Interviewer:** Thank you for sharing your experiences and coping strategies with us. Your insights will be invaluable in understanding the impact of Meningitis on individuals' lives.

**Participants 3:**

**Interviewer: Thank you for participating in this interview. Can you share your experience when you were affected by Meningitis?**

**Responses:** Certainly. It began with feeling unusually headache and neck pains. I actually thought I didn’t sleep well. However, it prolonged and was severe that, I was rushed to the hospital. I was admitted and spent about seven days undergoing treatment and rehabilitation. The whole family was terrified since it was live threatening.

**Interviewer: How did Meningitis change your daily life?**

**Responses:** I couldn’t do most of the things I use to do. As busy and active as I use to be, the sickness eventually literally made me dependent on my family for most of my needs Even water to bath, someone had to fetch me.

**Interviewer:** Can **you describe how your life has been affected overall?**

**Responses:** As it stands now, I cannot be like I use to be. Am now physically weak and will need to work with caution. I cannot sit continuously for long like I use to. I usually feel some pain at my back. I limit physical activities to my choice of work. My finances have also been affected. I spent a lot on medication at the hospital because my health insurance expired and am not able to work hard to improve my finances.

**Interviewer: What were the family roles you used to play then and now?**

**Responses:** As a mother and a house wife, I was directly in-charge of the house chores. I also use to brew pito that is quiet physical. Now, I have stopped brewing. Am considering cakes business at the market square since its less physical.

**Interviewer:** How did the condition affect your ability to work?

**Responses:** Am not the same person. Things I use to do, now I cannot do them. And like I mentioned anything physical, I try to minimize or restrict myself. And so, I am now selective with the kind of work I do.

**Interviewer: Have you noticed any changes in the attitudes of friends and family members toward you?**

**Responses:**Not so much is noticed. My family was very supportive especially when I was admitted. They visited, brought food and water among others. Even some friends were at the hospital to visit. Maybe there were some changes in attitude but I didn’t notice any to the best of my knowledge.

**Interviewer: Can you elaborate on how the condition affected your self-esteem?**

**Responses:** The sickness made me dependent on my family and I have lost my self-esteem. My work is not government work that I will still get paid when I was taken ill. I will have to work to earn the money. Unfortunately, following the sickness, I was not able to work to earn the money. I had to depend on my husband for almost everything. Basic ingredients such as salt and pepper I use to buy, am not able to do that. And sometimes I reach out to my siblings for support which am not too comfortable with.

**Interviewer: Moving on, can you share some coping strategies you employed when you were infected with the condition?**

**Responses:** I dependent heavily on my family especially my husband and my siblings to survive. Am organizing money to start cakes business at the make square. Am sure with the grace of God I will recover.

**Interviewer:** Thank you for sharing your experiences and coping strategies with us. Your insights will be invaluable in understanding the impact of Meningitis on individuals' lives.

Participants 4:

**Interviewer: Thank you for agreeing to participate in this interview. Let's start by discussing your experiences in managing a family member, friend, or neighbor who was infected with meningitis.**

**Responses:** Sure, I'm happy to share. When he was rushed to the hospital, one of the biggest challenges we faced was the financial burden. We didn’t have money anywhere, yet we had to pay bills. The cost of treatment, medications, and even feeding was quite expensive and put a significant strain on our finances. Additionally, managing his daily needs consumed all our time and energy, leaving little room for anything else.

**Interviewer: Can you elaborate on some of the socio-economic implications associated with managing or assisting the meningitis patient?**

**Responses:**The socio-economic implications were far-reaching. Aside the direct financial costs, there were indirect costs as well, such as lost wages due to time spent caring for the him transportation expenses to and from medical appointments, and the impact on productivity at work. For me, I will say it was pure sacrifice to support him amidst other social events such as funeral, work among others.

**Interviewer: What difficulties were encountered during treatment?**

**Responses:** There were several difficulties encountered during treatment. The lack of access to health facility within the community. Additionally, navigating the complexities of the healthcare system and understanding treatment options were all daunting tasks. Communication barriers with healthcare providers also posed significant challenge.

**Interviewer: How do you manage meningitis in this community?**

**Respondent:** Anybody sick here usually depending on the severity of the sickness may go to the hospital or self-medicate. Most people resort to self-medication and when they are not getting better, they then go to the hospital. Most times, it is at the health facility they inform us of the kind of sickness. We begin to hear meningitis around the warm season when you go to the hospital. We are usually told to report early to the hospital, educate community members about the signs and symptoms of meningitis, and the preventive measures.

**Interviewer: In your view, how can meningitis be effectively prevented in the community?**

**Responses:** Maybe vaccination should be widely available and accessible to all community members, especially children and the elderly. Health education campaigns should focus on raising awareness about the disease, its transmission, and preventive measures and early reporting to the hospital.

**Interviewer: Is there anything else you would like to share about your experiences in assisting the victim?**

**Respondent:** I just want to emphasize the importance of community support and solidarity in times of crisis. Managing meningitis can be overwhelming, but when we come together as a community to support one another, we can overcome even the most challenging circumstances.

**Participants 5**

**Interviewer: Thank you for participating in this interview. Let's discuss your experiences in managing a family member, friend, or neighbor who was infected with meningitis.**

**Responses:** Yes please. I'm happy to share my experiences.

**Interviewer:** What were some of the challenges you faced in managing the infected individual on a daily basis?

**Respondent:** One of the main challenges was the emotional toll it took on everyone involved. Seeing a loved one suffer with so much pain. Additionally, there were practical challenges such as ensuring she received proper medical care, managing her symptoms, and maintaining her comfort. Even though we had insurance, there were other cost we had to incur.

**Interviewer:** **Can you tell me about any socio-economic implications associated with managing or assisting the meningitis patients?**

**Responses:** Certainly. The socio-economic implications were significant. The cost of medical treatment, including hospitalization, medications, and doctor's visits, were financially burdensome for us. Additionally, often we take time off work to attend to her leading to lost of income and other opportunities. This exacerbated existing financial hardships on family members.

**Interviewer:** **What difficulties did you encounter during treatment?**

**Responses:** There were several difficulties during treatment. Access to healthcare services, especially specialized care for meningitis, in this rural area. This made it challenging to ensure timely and appropriate treatment. The pain she went through when they were taking the sample was unbearable. She still complains as at now when she sits for long. Psychological trauma was also unbearable for me as a mother.

**Interviewer:** **How is meningitis managed in your community?**

**Responses:** We go the hospital. My daughter was sent to the hospital when she was sick. The hospital is our first point of call.

**Interviewer:** **In your opinion, how can meningitis be effectively prevented in the community?**

**Responses:** Vaccinating all community members to prevent the spread. This can be supported with Health education campaigns to dispel myths and misconceptions about meningitis.

**Interviewer:** Is there anything else you would like to share about your experiences in assisting the infected individual?

**Responses:** No, am ok

**Participants 6**

**Interviewer: How has the condition changed your daily life?**

**Responses:** Meningitis completely upended my life as I knew it. Before, I was active, outgoing, and independent. Now, I find myself struggling with even the most basic activities of daily living. The symptoms of meningitis have made it incredibly difficult to maintain any semblance of normalcy. It's like I'm living in a fog, unable to fully engage with the world around me.

**Interviewer: Describe the family roles you used to play then and now.**

**Responses:** Before meningitis, I was the rock of my family. I took care of everyone, both emotionally and financially. But now, the tables have turned, and I find myself relying on them more than ever. They've had to pick up the pieces and take on roles they never imagined, while I struggle to come to terms with my new reality.

**Interviewer: Explain how the condition affected your ability to work.**

**Responses:**  Meningitis has had a devastating impact on my ability to work. I used to excel in my career, but now I struggle to even get through the day. The constant fatigue and brain fog make it nearly impossible to focus, let alone perform at the level I once did. It's incredibly frustrating to feel like I'm constantly falling behind, especially when I know what I'm capable of.

**Interviewer: How have friends' and family members' attitudes toward you changed?**

**Responses:**  Some have been incredibly supportive, standing by my side through thick and thin. But others seem to have a hard time understanding the severity of my condition. They expect me to bounce back to my old self, but that's just not possible. It's been a harsh lesson in who my true friends are and who I can rely on when times get tough.

**Interviewer: Can you elaborate on how the condition affected your self-esteem?**

**Responses:** Meningitis has shattered my self-esteem in ways I never could have imagined. I used to be confident in my abilities, but now I find myself doubting everything I do. It's hard not to feel worthless when you can't even perform simple tasks without feeling completely drained. The constant struggle with my own body has left me feeling defeated and powerless.

**Interviewer: How did emotional distress impact you and your family following your experience with meningitis?**

**Responses:**  It's difficult to put into words just how challenging that time was. Every day felt like a battle against my own body. The excruciating headaches, the overwhelming fatigue, and the constant sensitivity to light made every moment a struggle. Simple tasks that I once took for granted became monumental challenges, and I felt like I was living in a constant state of discomfort and fear.

**Interviewer: How can we support survivors effectively in their recovery?**

**Responses:** We can start by ensuring they have access to specialized medical care and rehabilitation services. Mental health support and counseling are also crucial. Building strong community support networks and advocating for policies prioritizing survivors' well-being are essential steps too.

Participants 7

**Interviewer: Tell me about your experience when you were predisposed to Meningitis.**

**Responses:** Okay, I'm not sure I'm comfortable with the question you are asking. Life has indeed been terrible following my discharge. Before I was infected with the condition, I was pregnant and had to be detained in the hospital for almost two months of hard stress and intensity. I was finally discharged, came home, and always felt weak and dizzy. I eventually went into labor, suffered in there, only for my husband to come in and the doctor telling me that the baby wasn't staying and they needed to save me and the baby. So, the doctor said he wanted to run some procedures. This wasn't well communicated to me, and I consented only when I recovered, and they were telling me my womb was turned so that I cannot give birth. This made me feel bad till date. How can a woman's womb be blocked? Was it because of the sickness that made them block my womb? What happens if my husband should divorce me? Where do I have to start from? In fact, this CSM has really caused me a lot of pain, and I can never forget that till I die.

**Interviewer:** Has **the condition changed your daily life in any way?**

**Responses:** What else do you want me to say ….. It's turned my life upside down. Before all this, I was looking forward to welcoming my baby into the world, but now I'm left grappling with physical and emotional scars that may never fully heal. I feel like I've lost a part of myself, and I'm struggling to find my footing in this new reality.

**Interviewer:** Describe **the family roles you used to play then and now.**

**Responses:** Before meningitis, I was a wife, a mother-to-be, and a caretaker for my family. Now, I feel like I'm barely holding on. My husband has had to take on more responsibilities, and I can't help but feel like I'm letting everyone down. It's a constant battle between wanting to be there for my family and struggling to take care of myself.

**Interviewer: Explain how the condition affected your ability to work.**

**Responses:** That feels like a distant memory now. I used to have a job, dreams, ambitions. But now, just getting out of bed in the morning feels like an insurmountable task. The physical and emotional toll of meningitis has left me feeling drained and defeated. I don't know if I'll ever be able to return to the person I once was.

**Interviewer: How did emotional distress impact you and your family following your experience with meningitis?**

**Responses:** The emotional distress has been overwhelming, both for me and my family. Before meningitis, I was full of hope and excitement about welcoming a new life into the world. But the trauma of being hospitalized, enduring a difficult labor, and then discovering that I couldn't conceive again has left me shattered. I've been grappling with feelings of grief, anger, and confusion, while also worrying about the impact on my family. It's been a rollercoaster of emotions, and some days, it feels like I'll never find peace.

**Interviewer: How do you think these challenges can be addressed to support survivors in their recovery journey?**

**Responses:** Addressing these challenges requires a comprehensive approach that acknowledges the physical, emotional, and psychological toll of meningitis. Firstly, survivors need access to specialized medical care and rehabilitation services to help them cope with the physical limitations and regain their strength. Additionally, providing mental health support and counseling is crucial in helping individuals navigate the complex emotions and trauma associated with the illness. Creating support networks within the community can also provide a sense of belonging and solidarity, reducing feelings of isolation and stigma. Ultimately, raising awareness about the long-term effects of meningitis and advocating for policies that prioritize survivor's well-being are essential steps in supporting their recovery journey.

Participants 8

**Interviewer:** Tell me about your experience when you were predisposed to Meningitis.

I am a new person following my survival from this sickness. I now can’t socialize like I used to do, I sit as if I have nobody in my life. I have five children all of them too are in the south. It has come to be like, if not my women group members who sometimes come to visit, my only interaction rests on my husband who is equally not the talking type. In fact, this sickness reminds me of a proverb by my grandfather who says that “it is only when you are confronted with a problem that you know your loved ones” but this sickness has really brought me to the reality of the proverb.

**Interviewer:** Has **the condition changed your daily life in any way?**

**Responses:** Yes. Before meningitis, I was active in my community, always surrounded by friends and family. Now, I feel isolated and alone. My energy levels are low, and I struggle to engage in activities that once brought me joy. It's like I'm living in a different world, one where I don't quite belong anymore.

**Interviewer:** Describe **the family roles you used to play then and now.**

**Responses:** Before meningitis, I was the matriarch of my family, the one everyone turned to for guidance and support. Now, I feel like I'm barely keeping it together. My children are far away, and I miss them terribly. I used to be the one who held everything together, but now I feel like I'm falling apart.

**Interviewer:** Explain **how the condition affected your ability to work.**

**Responses:** of bed in the morning feels like an impossible task. The fatigue and weakness from meningitis have taken a toll on my body, and I don't know if I'll ever fully recover. It's hard not to feel useless and defeated.

**Interviewer: How have friends' and family members' attitudes toward you changed?**

**Responses:** Some have been supportive, understanding of the challenges I'm facing. But others seem to expect me to just snap out of it, as if meningitis is something you can just shake off. It's frustrating and disheartening, to say the least. I wish they could see the invisible battle I'm fighting every day and offer more compassion and empathy.

**Interviewer: Can you elaborate on how the condition affected your self-esteem?**

**Responses:** Meningitis has definitely taken a toll on my self-esteem. I used to feel confident and capable, but now I feel like a shadow of my former self. The physical and emotional toll of the illness has left me feeling weak and vulnerable, and it's hard not to let that affect how I see myself. I'm trying to stay positive, but some days it feels like an uphill battle.

Participant 9

**Participant 9: Interviewer: When your family member, friend, or neighbor was infected with meningitis, what were some of the challenges you faced in managing him/her daily?**

**Responses:** Family support was massive. If not for my family, I would have died. I don’t really have friends like that at home because am not based here. If it was Afram Plains, I could tell their level of support. Financial pressure on the household following the incidence was immense. The incident happened at a time when we didn’t have money at home. I was in the farm when I was called that our daughter fell and could not walk. I wasn’t having a motorbike. I bought fuel into someone’s motor and we rushed her to Jirapa hospital where she was detained for three months. During her admission, lab tests were run and all this was costly; it wasn’t easy. We could buy medicine for two three times before the day will end. Hmmmmmmm……we were actually not laughing there. We were financially down, but we had no choice because our daughter’s life was at stake.

**Interviewer: What were some of the socio-economic implications associated with managing or assisting the meningitis patients?**

**Responses:** It is important to recognize that family support is not limited to the immediate aftermath of the illness but extends throughout the entire recovery process. Meningitis comes along with long-term effects on the patient's physical and cognitive abilities, and ongoing family support helps the individual navigate these challenges and adapt to any changes in their daily lives. If not for our support, my brother would have passed on by now. We had to rush him to the hospital, and we have since been around encouraging him morally and physically supporting him. Following our brother's recovery, we were happy. However, we now struggle to put food on the table because we have almost depleted the little we were depending on in order to cover the expenses of medical bills and daily transportation of food to the hospital. The situation has led to significant indebtedness in our household, to the point that we are not trusted within the community. We had to resort to borrowing to fulfill our financial obligations and meet other needs.

**Interviewer: What difficulties were encountered during treatment?**

**Responses:** The difficulties encountered during treatment were mainly related to the long-term effects of meningitis on the patient's physical and cognitive abilities. These effects can vary widely depending on the severity of the illness and the individual's overall health, but they often include issues such as fatigue, weakness, difficulty concentrating, and memory problems.

**Interviewer: How do you manage meningitis in this community?**

**Responses:** In our community, we manage meningitis by ensuring that patients receive prompt medical attention and follow-up care, as well as providing them with ongoing support and assistance as they recover. This may include helping them with daily activities, providing emotional support, and encouraging them to participate in rehabilitation programs to help them regain lost abilities.

**Interviewer: In your view, how can meningitis be effectively prevented in the community?**

**Responses:** Meningitis can be effectively prevented in the community through measures such as vaccination, good hygiene practices, and early detection and treatment of cases. Vaccination programs can help prevent the spread of meningitis by ensuring that individuals are protected against the most common strains of the disease. Additionally, practicing good hygiene, such as washing hands regularly and avoiding close contact with sick individuals, can help reduce the risk of infection. Early detection and treatment of meningitis cases are also crucial for preventing the spread of the disease and minimizing its impact on affected individuals and communities.

**Interviewer: Is there anything else you would like to share about your experiences in assisting the victim?**

**Responses:** I just want to emphasize the importance of family support in helping individuals recover from meningitis. Our support doesn't end when the patient is discharged from the hospital; it continues throughout the entire recovery process and beyond. By providing ongoing support and assistance, we can help our loved ones navigate the challenges of meningitis and move forward with their lives.

**Participant 10:**

**Interviewer: Can you describe some of the challenges faced by survivors of meningitis in performing daily tasks?**

**Responses:** Survivors of meningitis in the Upper West Region faced significant challenges that limited their ability to perform hard tasks especially my senior brother sons down times. These challenges encompassed physical and cognitive disabilities, as well as emotional distress. For instance, one participant shared, "It has really not been easy. I was actually processing shea butter to sell for a living as well as selling 'Koosee' and assisting my husband to farm, but now the strength is not there. I can no longer bend down for long because of waist pain. So, the quantity of work I used to do has drastically reduced, hence affecting my productivity and source of livelihood."

**Interviewer: How did the impact of meningitis affect the ability of individuals to perform major tasks?**

**Responses:** The impact of meningitis weighed heavily on the patient’s ability to perform major tasks. One aunty explained, "The condition represented a significant barrier in his daily life, affecting his productivity, independence, and overall sense of well-being. He was virtually farming to feed us; unfortunately, he went down completely and could not support us in farming again, even though he recovered. His outcome has drastically changed. Someone who used to farm and would yield close to 50 bags of maize and around 10 bags of groundnut is now struggling to produce even a single bag of maize, let alone groundnut. In fact, we have suffered significant losses..."

**Interviewer: How did emotional distress impact the survivors and their families?**

**Responses:** Emotional distress resulting from meningitis profoundly affected survivors and their families. An aunty of as urvisor shared that , "My son's happiness has disappeared since he was deeply affected by meningitis. Now, when two or more people are sitting together, he prefers to isolate himself and just watch. This has alone put the family in a very disorganized state. Currently, we simply don't know what pleases him. Life!" The emotional toll was also articulated by a survivor who added, "It's been a journey, you know? Dealing with cognitive hiccups feels like my own mind playing tricks. The emotional toll? Heavy frustration, self-doubt, it's all there. Now, the harder tasks aren't just tasks; they're massive obstacles. Not that I don't want to tackle them, but cognitive glitches team up with emotional baggage, making even simple things feel like climbing a mountain. So, yeah, grappling with this cognitive-emotional task constraint."

**Interviewer: How do you think these challenges can be addressed to support survivors in their recovery journey?**

**Responses:** During the season, we need to seek prompt check when we suspect any sickness that has the high temperature, once neck turning backward, once four head aching etc

Prompt reporting to the health facility is another way to saving patients of CMC. If the because of nurse Ajara seek who came and rushed my brothers son to the hospital, like by now my son wouldn’t have had story to say today. His alive and kicking with his daily life.

We are advice to sleep in an environment and room. We should crowd ourselves in one room. Once people crowd themselves in one room. There will be breathing on each other and this may trigger the spread of the disease.

We should also clean our room, environment, bowl and eat good food.

Participants 11

**Interviewer: When your family member, friend, or neighbor was infected with meningitis, what were some of the challenges you faced in managing him/her daily?**

**Responses:** Managing a loved one with meningitis was incredibly challenging. Beyond the financial strain of medical expenses, there was the emotional toll of watching them suffer. Each day presented new obstacles, from ensuring they received proper care to balancing our own responsibilities and commitments.

**Interviewer: What were some of the socio-economic implications associated with managing or assisting the meningitis patients?**

**Responses:** The socio-economic implications were profound. We faced unexpected medical costs and had to reorganize our finances to cover them. This often meant sacrificing other expenses or even taking time off work to provide care, resulting in a loss of income for our family.

**Interviewer: What difficulties were encountered during treatment?**

**Responses:** Treatment posed numerous challenges. From logistical issues like arranging transportation to medical appointments to the emotional strain of witnessing our loved one's suffering, every aspect of the treatment process was fraught with difficulty. It required immense patience, resilience, and support from our community.

**Interviewer: How do you manage meningitis in this community?**

**Responses:** In our community, managing meningitis involves a multi-faceted approach. We prioritize prevention through education and vaccination campaigns, emphasizing the importance of early detection and prompt medical intervention. Additionally, we provide support and resources to affected families, fostering a sense of solidarity and resilience.

**Interviewer: In your view, how can meningitis be effectively prevented in the community?**

**Responses:** Effective prevention of meningitis requires a combination of vaccination programs targeting vulnerable populations, promoting good hygiene practices, and raising awareness about the symptoms of the disease. By empowering individuals to take proactive measures and seek medical help when needed, we can mitigate the impact of meningitis in our community.

**Interviewer: Is there anything else you would like to share about your experiences in assisting the victim?**

**Responses: I** want to underscore the importance of community support during times of crisis. By coming together to support those affected by meningitis, we not only alleviate their burden but also strengthen the fabric of our community. Compassion, empathy, and solidarity are essential in navigating the challenges posed by meningitis and other health crises.

**Participants 12**

**Interviewer:** **When your family member, friend, or neighbor was infected with meningitis, what were some of the challenges you faced in managing him/her daily?**

**Responses:** The hustle of commuting daily to the hospital, paying for medication and my continuous absence from my work side attracted wrath of my boss.

**Interviewer:** **What were some of the socio-economic implications associated with managing or assisting the meningitis patients?**

**Responses:** It was really challenging with the hospital bills and some indirect cost. We had to borrow to settle the bills even though it wasn’t easy. About two people after listening to my story said they didn’t have the money. It was really humiliating.

**Interviewer:** **What difficulties were encountered during treatment?**

**Responses:** When we got the hospital, they were initially not telling me anything and I was restless. They later referred us to the hospital. We struggled to get a tricycle that took us to the hospital. I felt there was an undue delay in responding to my client. My client was also traumatized.

**Interviewer:** **How do you manage meningitis in this community?**

**Responses:** We go to the health center here. And sometimes like my case we are referred to the hospital.

**Interviewer:** **In your view, how can meningitis be effectively prevented in the community?**

**Responses:** Through public sensitization on the causes, symptoms and how to prevent meningitis.

**Interviewer:** **Is there anything else you would like to share about your experiences in assisting the victim?**

**Responses:** No except to say that our health center should be well resourced to help us.

Participants 13

**Interviewer:** When your family member, friend, or neighbor was infected with meningitis, what were some of the challenges you faced in managing him/her daily?

**Responses:** The psychological trauma, running round all alone and financial burden in taking care of her were some of the challenges. I remember I stepped out to get something and upon my return, I noticed that they used the screen round her. I screamed thinking she was dead. Fortunately, I was told they were taking sample from her.

**Interviewer:** What were some of the socio-economic implications associated with managing or assisting the meningitis patients?

**Responses:** I was affected socially and economically. I sell in the community daily market. Since she was taking ill, haven’t been able to open my shop to sell anything. That is also my only source of money. Most of our village savings and loans meetings, I couldn’t attend among other events.

**Interviewer:** What difficulties were encountered during treatment?

**Responses**: I was the only one running round to attend to her. I eventually had to call my sister to sometimes cook or take care of her whiles I also take some rest. So yes, I was overwhelmed during that period and almost became a patient myself. I was also worried about her school. She was admitted whiles her colleagues were still in school.

**Interviewer:** **How do you manage meningitis in this community?**

**Responses**: We go to the hospital. Right away, when my daughter came home, I immediately sent her to the hospital and now she is ok. We are also reminded of the safety protocols by the healthcare workers anytime we are in the meningitis season.

**Interviewer:** **In your view, how can meningitis be effectively prevented in the community?**

**Responses:** Avoid overcrowded areas and sleeping in a well-ventilated area. They used to vaccinate but now we don’t see that again. If they could also continue with the vaccination would have been helpful. Also report early to the hospital when you are not well.

**Interviewer:** **Is there anything else you would like to share about your experiences in assisting the victim?**

**Responses:**  The health education by healthcare workers should start early and not when we begin to report cases.

Participant 14

**Interviewer:** Tell me about your experience when you were predisposed to Meningitis.

**Response:** My brother, it wasn’t a pleasant experience at all. It was a battle each day going through the pain. I was scared and thought I was going to die. I particularly struggled because it was not detected early. When I was not well, I didn’t immediately visit the hospital, unfortunately it aggravated.

**Interviewer:** How has the condition changed your daily life?

**Responses:**  Once you are sick you are not able to do anything particular when the situation aggravates. I was indisposing and basically had to depend on family when I was admitted. However, even though am not too well, as the bread winner had to adjust to get back to work.

**Interviewer:** Describe the family roles you used to play then and now.

**Responses:** I am the bread winner for the family. There haven’t been much change. Temporally, my wife was there to support but am still the one running the house although under difficult circumstance.

**Interviewer:** Explain how the condition affected your ability to work.

**Responses:** Temporally, I was indisposed, especially when I was admitted. However, as the bread winner of the family, I had to adjust and work.

**Interviewer:** How have friends' and family members' attitudes toward you changed?

**Responses:**  No, not at all. Like I mentioned, my family was there for me.

**Interviewer:** Can you elaborate on how the condition affected your self-esteem?

**Responses:** My self-esteem was not affected since I was still the one providing even though I was down.

Participants 15

**Interviewer:** **Tell me about your experience when you were predisposed to Meningitis.**

**Responses:**  Okay. I was travelling from down south coming home for a funeral. What I can remember is that I started sweating profusely. I later woke up at the hospital. I was told I went unconscious when they got to Nandom and they drove me straight to the hospital. I woke in fear and under intense trauma.

**Interviewer:** **Has the condition changed your daily life in any way?**

**Responses:**  hmmmmmmm. I was based down south with my family and now my children say I shouldn’t come again. I didn’t plan to stay here however, following the sickness, they are remained here. I do nothing here and basically depend on them to send me money to survive on.

**Interviewer:** **Describe the family roles you used to play then and now.**

**Responses:** I fend for myself and my wife with the basic needs including feeding. I farm on my own to take care of our needs. My children are all grown and insisted I stop farming. However, this keeps me active and less dependent on them. I have lived over 20 years in Techiman and now coming home unprepared is worrying to say the least.

**Interviewer:** **Explain how the condition affected your ability to work.**

**Responses:**  It has affected me seriously as I explained. I don’t even have a farm to farm on now even though am not completely healed.

**Interviewer:** **How have friends' and family members' attitudes toward you changed?**

**Responses:** The family back home respondent promptly when I gained consciousness. In fact, they have been there for me. Even though my immediate family is not here, my siblings were very supportive. I don’t really have friends here since am not based here.

**Interviewer:** **Can you elaborate on how the condition affected your self-esteem?**

**Responses:** Now that I depend on my family for everything I will be seen as a bother. If am not given money, I won’t be able to do anything.
